# Atmospheric Pressure Plasma Polymerized Oxazoline-Based Thin Films—Antibacterial Properties and Cytocompatibility Performance

**DOI:** 10.3390/polym11122069

**Published:** 2019-12-12

**Authors:** Pavel Sťahel, Věra Mazánková, Klára Tomečková, Petra Matoušková, Antonín Brablec, Lubomír Prokeš, Jana Jurmanová, Vilma Buršíková, Roman Přibyl, Marián Lehocký, Petr Humpolíček, Kadir Ozaltin, David Trunec

**Affiliations:** 1 Department of Physical Electronics, Faculty of Science, Masaryk University, Kotlářská 2, 611 37 Brno, Czech Republic; pstahel@physics.muni.cz (P.S.); abr92@sci.muni.cz (A.B.); luboprok@gmail.com (L.P.); janar@physics.muni.cz (J.J.); vilmab@physics.muni.cz (V.B.); 451677@mail.muni.cz (R.P.); 2 Faculty of Chemistry, Institute of Physical and Applied Chemistry, Brno University of Technology, Purkyňova 118, 612 00 Brno, Czech Republic; mazankova@fch.vut.cz (V.M.); ktomeckova11@gmail.com (K.T.); 3 Department of Mathematics and Physics, Faculty of Military Technology, University of Defence in Brno, Kounicova 65, 662 10 Brno, Czech Republic; 4 Faculty of Chemistry, Institute of Food Science and Biotechnology, Brno University of Technology, Purkyňova 118, 612 00 Brno, Czech Republic; matouskova@fch.vut.cz; 5 Centre of Polymer Systems, Tomas Bata University in Zlín, Trida Tomase Bati 5678, 760 01 Zlín, Czech Republic; lehocky@post.cz (M.L.); humpolicek@utb.cz (P.H.); kadirozaltin@hotmail.com (K.O.)

**Keywords:** antibiofouling, plasma polymer, oxazoline

## Abstract

Polyoxazolines are a new promising class of polymers for biomedical applications. Antibiofouling polyoxazoline coatings can suppress bacterial colonization of medical devices, which can cause infections to patients. However, the creation of oxazoline-based films using conventional methods is difficult. This study presents a new way to produce plasma polymerized oxazoline-based films with antibiofouling properties and good biocompatibility. The films were created via plasma deposition from 2-methyl-2-oxazoline vapors in nitrogen atmospheric pressure dielectric barrier discharge. Diverse film properties were achieved by increasing the substrate temperature at the deposition. The physical and chemical properties of plasma polymerized polyoxazoline films were studied by SEM, EDX, FTIR, AFM, depth-sensing indentation technique, and surface energy measurement. After tuning of the deposition parameters, films with a capacity to resist bacterial biofilm formation were achieved. Deposited films also promote cell viability.

## 1. Introduction

Polyoxazolines (POx) are a promising and important class of polymers that have attracted substantial attention recently due to their antibiofouling properties [1,2] and good biocompatibility [3]. Usually, POx are prepared by living-cationic ring-opening polymerization, which is a lengthy wet process conducted in organic solvents. POx thin films are created in a subsequent step, which needs to be tailored for any particular type of substrates [4,5]. Other possible polymerization techniques are photocoupling [6] and grafting [7], both of which require the premodification of substrates. So, the formation of polyoxazoline coatings using conventional methods is a slow and complex multistep procedure, which can be conducted only on a limited range of substrates. The difficulties of these conventional methods can be overcome via plasma polymerization. Plasma polymerization is known to be a suitable method for the deposition of many biomaterial coatings [8,9]. Plasma polymerization is in the class of plasma-enhanced chemical vapor deposition (PECVD) methods, which are successfully used for example for thin film deposition [10], surface modification [11,12], or growing of nanomaterials [13,14,15]. Plasma deposition of 2-methyl-2-oxazoline and 2-ethyl-2-oxazoline has already been performed in low-pressure radio frequency (RF) discharge [16,17,18]. However, the necessity to use expensive vacuum pumping systems is the disadvantage of low-pressure plasma deposition techniques. Recently, plasma deposition in atmospheric pressure discharges has become a new promising technology due to its economic and ecological advantages. A suitable discharge type for plasma deposition at atmospheric pressure is a homogeneous dielectric barrier discharge (DBD), which can be obtained in nitrogen. This discharge type is called atmospheric pressure Townsend-like discharge (APTD) [19]. APTD in the mixture N${}_{2}$–SiH${}_{4}$–N${}_{2}$O was used for SiO${}_{2}$ thin film deposition [20], APTD in the mixture of N${}_{2}$ with hexamethyldisiloxane (HMDSO) was used for deposition of organosilicon polymer films [21]. The properties of films deposited in APTD are different from properties of films deposited in low-pressure discharges due to different plasma and discharge parameters, e.g., ion energies in APTD are lower than those in low pressure discharges. The film properties can be changed by increasing the substrate temperature at deposition [22]. Recently, POx coatings were deposited using discharges at atmospheric pressure. Atmospheric pressure helium plasma jet was used for plasma polymerization of 2-methyl-2-oxazoline on heated silicon substrates [23]. The film stability in buffer solution was substantially improved when the films were deposited at substrate temperatures above 50 ${}^{\circ }$C in these experiments. Near atmospheric pressure (0.5 bar) plasma polymerization of 2-alkyl-2-oxazolines in argon DBD was used for the study of the influence of the aliphatic side-chain length on the plasma polymerization process conditions as well as on the properties of the deposited coatings [24].

In the current study, POx thin films were deposited in nitrogen APTD using 2-methyl-2-oxazoline as monomer. The substrate temperature was changed from 20 ${}^{\circ }$C to 150 ${}^{\circ }$C at the film deposition. This temperature change leads to different film properties. The films with the highest biocompatibility and the best antibiofoulding properties were obtained at the deposition with a substrate temperature of 150 ${}^{\circ }$C.

## 2. Materials and Methods

### 2.1. Materials

Glass plates (soda-lime glass, 150 × 100 mm, thickness 1.1 mm) were used as substrates for deposition. 2-Methyl-2-oxazoline (98%, Sigma-Aldrich, Munich, Germany) was used as a monomer for plasma deposition. Preliminary antibacterial tests were done with *Staphyloccocus epidermidis* (CCM 4418), supplied by the Czech Collection of Microorganisms in Brno. Bacterial culture was grown into commercial BHI medium (Brain Heart Infusion Broth, HiMedia, Mumbai, India). Basic Red 2 (Safranin O, Sigma-Aldrich, Munich, Germany) stain was used for biofilms visualization. Other antibacterial tests were done with *Staphylococcus aureus* (CCM 4516) and *Escherichia coli* (CCM 4517), both supplied by the Czech Collection of Microorganisms in Brno.

### 2.2. Plasma Deposition

**Figure 1:**
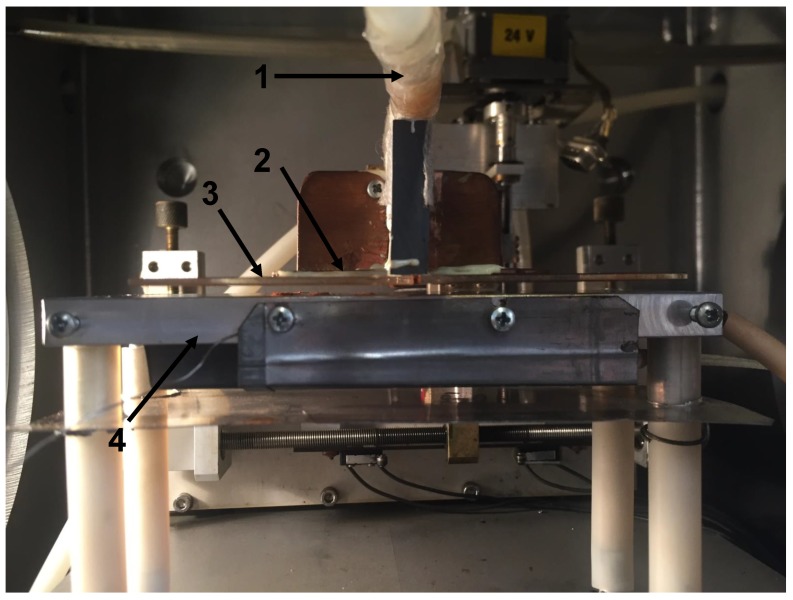
The image of electrode system. 1—gas inlet; 2—upper electrode; 3—glass; 4—lower heated electrode (without substrate).

Plasma polymerization was performed in a custom-built reactor (metallic chamber with dimensions 500 mm × 500 mm × 500 mm) with dielectric barrier discharge [22]. The discharge was generated between two planar metal electrodes. The bottom electrode with dimensions 150 mm × 55 mm could be heated using a heating spiral, and the electrode temperature was measured with a thermocouple. The upper electrode with dimensions 55 mm × 40 mm was covered with glass, 1.5 mm in thickness. A slit 2 mm wide in the center of the upper electrode was used for the supply of working gas with the monomer to the discharge. The electrode system is shown in Figure 1.

The scheme of the electrode system can also be found in a previous study [25], where gas flow in the discharge gap was also modeled. The glass substrates were cleaned in a mixture of cyclohexane and isopropyl alcohol (1:1) and dried in airflow. Clean substrates were put into the reactor on the bottom electrode, which was then entirely covered by the substrate. The discharge gap between the substrate and the upper electrode was set to be 1.0 mm. Before starting the depositions, the discharge chamber was pumped down to a pressure of 100 Pa and then filled with nitrogen to a pressure of 101 kPa. Atmospheric pressure during the deposition was maintained by slight pumping.

Nitrogen flow with flow rates from 50 sccm to 400 sccm bubbled through the liquid 2-methyl-2-oxazoline monomer in a glass bottle container. This flow was then mixed with the main nitrogen flow with the flow rate of 500 sccm. The temperature of the monomer was kept constant and set to 20 ${}^{\circ }$C. The monomer flow rate was determined by weighing the monomer before and after the deposition, the flow rate of the monomer was 20 mg s${}^{-1}$ for the nitrogen flow of 100 sccm through the liquid monomer. A high voltage with a frequency 6 kHz was used for the generation of a dielectric barrier discharge in the discharge gap. The discharge was burning in homogeneous APTD mode [21], which is suitable for the deposition of homogeneous thin films. The discharge mode was checked by current–voltage measurements using an oscilloscope. The input power to a high voltage source was set to 55 W and kept constant in all experiments presented in this paper. The temperature of the bottom electrode was increased to a given value before the deposition. The upper electrode was periodically moving with a speed of 0.6 cm s${}^{-1}$ above the substrate during the deposition in order to ensure an even greater homogeneity of the deposited film. The deposition time was 23 min.

### 2.3. Surface Characterization

Deposited films were imaged with scanning electron microscope (SEM) MIRA3 (TESCAN, Brno, Czech Republic) with a Schottky field emission electron gun equipped with secondary electron and back-scattered electron detectors as well as a characteristic X-ray detector (EDX) analyzer (Oxford Instruments, High Wycombe, UK). The IR spectra of deposited films were measured by FTIR spectrometer Alpha (Bruker, Billerica, MA, USA) using a single reflection ATR module Platinum. The total surface free energy of the films was determined from measurements of contact angles between testing liquids and the film surfaces using a sessile-drop technique. Acid-base theory was used for the calculation of total surface free energy. Atomic Force Microscope (AFM) Ntegra Prima (NT-MDT, Apeldoorn, The Netherlands) was used to study the surface topography of the POx films. The measurements were performed in semicontact mode on 10×10 μm${}^{2}$ and 5×5 μm${}^{2}$ areas of each coating with a scanning rate of 0.5 Hz. The 3D roughness parameters of the thin films were evaluated according to the ASME B46 standard using the NovaPx software (NT-MDT). The film thickness was measured using a Dektak XT (Bruker, Tucson, AZ, USA) mechanical profilometer.

### 2.4. Characterization of Mechanical Properties

Hardness (*H*) and effective elastic modulus ($E_{\mathrm{eff}}$) of POx films were assessed by a nanoindentation tester Hysitron TI 950 TriboIndenter (Bruker, Minneapolis, MN, USA) with a load resolution of 1 nN. The effective elastic modulus could be expressed according to $E_{\mathrm{eff}}=E/\left(1-\nu^{2}\right)$, where ν and *E* are the Poisson’s ratio and Young’s modulus of the material, respectively. The standard Oliver and Pharr method [26] was used to calculate the above listed parameters. For a reliable characterization of mechanical properties of POx films several indentation modes were used including basic quasistatic indentation tests with trapezoid load function (5 s loading, 2 s creep, 5 s unloading), quasistatic nanoindentation tests with 33 partial unloading segments, and nanodynamic indentation (nanoDMA) in constant strain-rate measuring mode. The nanoDMA indentation tests were carried out by superimposing a sinusoidal load with a small amplitude (30 μN to 0.2 mN) and a frequency of 220 Hz on the quasistatic loading curve. From the sample response to this dynamic loading force, the dynamic displacement amplitude and the phase shift ϕ between this dynamic displacement and the input sinusiodal loading force were acquired. In order to decouple the indenter and the sample contribution from the the total spring stiffness, *k*, and the total damping coefficient, *C*, the Kelvin–Voigt mechanical equivalents model was used [27]. In this model, the indenter and the sample are described by a viscous damper and a purely elastic spring, which are connected in parallel. On the basis of the above described method, the storage modulus $E^{\prime }$, loss modulus $E^{\prime \prime }$, and loss factor tanϕ were calculated. Equation (1) shows the relationship between $E^{\prime }$, $E^{\prime \prime }$ and complex modulus (denoted as $E^{*}$):

(1) $$E^{\prime }=\frac{k_{s}\sqrt{\pi }}{2\sqrt{A_{c}}};\;E^{\prime \prime }=\frac{\omega C_{s}\sqrt{\pi }}{2\sqrt{A_{c}}};\;E^{*}=E^{\prime }+iE^{\prime \prime }\;,$$

where *i* is the imaginary unit, $A_{c}$ is the projected contact area between the tip and the sample, ω is the angular frequency of the oscillating load, $k_{s}$ is the sample spring stiffness, and $C_{s}$ is the sample damping coefficient.

The maximum indentation load was varied between 100 μN and 11 mN. The mechanical parameters were determined at indentation depths <1/10 of film thickness to avoid any substrate effect.

### 2.5. Antibacterial Tests

Bacterial culture *Staphyloccocus epidermidis* was grown into brain heart infusion (BHI) medium, and the temperature for microorganism cultivation was 37 ${}^{\circ }$C. After 24 h incubation, *S. epidermidis* was diluted with new sterile medium to $1\times 10^{8}$ CFU per mL based on turbidity (NanoPhotometer™ P300, Implen, Munich, Germany). Then, *S. epidermidis* suspension (500 μL) was added on each sample. The bacteria on the surface of the samples were incubated for 24 h to allow the formation of biofilms. After incubation, all samples were washed twice with Milli-Q water to remove any loosely bound biofilm. For biofilms visualization, 200 μL of Basic Red 2 stain was used. The excess of Basic Red 2 stain was then washed off and the results were evaluated using optical microscopy (Optical microscope Intraco Micro LM 666 PC/*∞* with Dino-Capture 2.0 software, Tachlovice, Czech Republic). Samples were imaged at least fifteen times each at random points, and the surface area covered by bacteria was quantified using images.

Other antibacterial tests were performed according to the ISO 22196 procedure with modifications. Before antibacterial testing, samples were sterilized by UV–radiation (wavelength of 258 nm) for 30 min. For the determination of antibacterial performance, gram-positive *Staphylococcus aureus* and gram-negative *Escherichia coli* were used. Bacterial suspensions (*E. coli* $3.4\times 10^{6}$ CFU mL${}^{-1}$; *S. aureus* $8.9\times 10^{5}$ CFU mL${}^{-1}$) were prepared in 1/500 Nutrient broth (HiMedia laboratories, Mumbai, India). The bacterial suspension was dispensed on the sample surface (dimensions 25 mm × 25 mm) in the volume of 100 μL and the sample was covered with the polypropylene foil (dimensions 20 mm × 20 mm). Samples with foils were cultivated at 35 ${}^{\circ }$C and 100% relative humidity for 24 h. After the incubation time, polypropylene foil was removed and each sample was completely washed by SCDLP (Soybean, Casein, Digest, Lecithin, Polysorbate) broth (HiMedia laboratories, India), which was subsequently collected. The viable bacteria count was determined by the pour plate culture method (PCA, HiMedia laboratories, India).

### 2.6. Cytocompatibility Test

The mouse embryonic fibroblast continuous cell line (NIH/3T3, ATCC^®^ CRL-1658™, Teddington, UK) was used for cytocompatibility test, according to the EN ISO 10993-5 standard, with modification. As a culture medium, the ATCC-formulated Dulbecco’s Modified Eagle’s Medium (BioSera, Nuaille, France), containing 10% calf serum (BioSera, France) and Penicillin/Streptomycin at 100 U mL${}^{-1}$ (PAA Laboratories GmbH, Pasching, Austria) was used. The tested samples were prepared with a dimension of 10 mm × 10 mm and sterilized by UV–radiation (wavelength of 258 nm) for 30 min and placed into the 24 well-plate. The cells were seeded onto the samples in the concentration of $1\times 10^{4}$ for an hour for adhesion of the cells. After the precultivation, a sufficient amount of the medium was added and incubated for 72 h at 37 ${}^{\circ }$C. The changes in cell morphology were observed with an inverted fluorescent microscope (Olympus, IX 81). In order to assess the cytotoxic effect, an MTT assay (Duchefa, Biochemie, Haarlem, The Netherlands) was performed. The absorbance was measured by an Infinite M200 Pro NanoQuant absorbance reader (Tecan, Männedorf, Switzerland). All tests were performed three times.

## 3. Results

The POx films were deposited at substrate temperatures 60 ${}^{\circ }$C, 90 ${}^{\circ }$C, 120 ${}^{\circ }$C, 150 ${}^{\circ }$C, and at nitrogen flow of 100 sccm through the monomer. It was found that the POx films deposited at substrate temperatures up to 90 ${}^{\circ }$C can be washed by water from the substrate. Similar results were also observed in a study by other authors [18], where the POx films deposited at low RF power supplied to RF discharge were also soluble in water. So, films deposited at the substrate temperature of 150 ${}^{\circ }$C will be characterized in the following subsections. The changes of nitrogen flow rate through the monomer influenced the film thickness only, so the depositions were performed with a nitrogen flow of 100 sccm.

### 3.1. Surface Characterization

**Figure 2:**
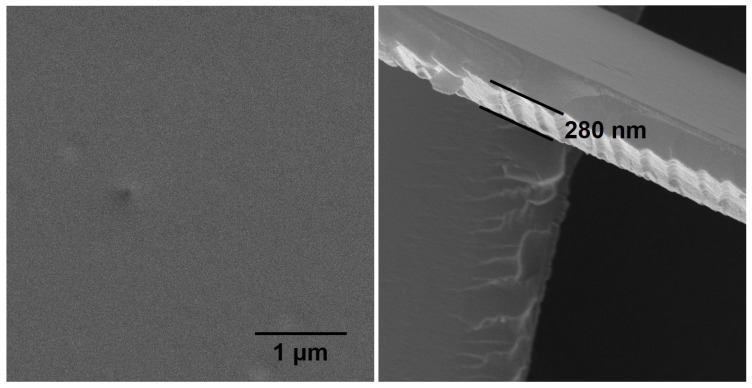
SEM images of polyoxazoline (POx) film deposited at 150 ${}^{\circ }$C. Left images—surface of deposited film, magnification 10k×; right image—deposited film removed from the substrate by scratch, magnification 50k×.

The image from SEM of films deposited at 150 ${}^{\circ }$C is shown in Figure 2. It can be seen from this image (and other images) that deposited films are smooth and without any pinholes. The elemental composition of POx films deposited at different substrate temperatures determined by EDX is shown in Table 1.

The nitrogen content in the films was increased by up to 40% in comparison with nitrogen content in 2-methyl-2-oxazoline. The oxygen content corresponds to oxygen content in 2-methyl-2-oxazoline at substrate temperatures of 60 ${}^{\circ }$C and 90 ${}^{\circ }$C and it decreases to lower values at substrate temperatures of 120 ${}^{\circ }$C and 150 ${}^{\circ }$C. Additionally, carbon content increases at higher substrate temperatures.

The contact angles between the test liquids and POx films were measured in order to determine the total surface free energy using the sessile drop technique. Three test liquids were used—distilled water, glycerol, and diiodomethane (CH${}_{2}$I${}_{2}$). The acid–base theory with multiple regression [28] was used to calculate the total surface free energy and its components—the Lifshitz–van der Walls (LW) interaction component and the acid–base (AB) interaction component. The surface free energy and its abovementioned components of POx films are given in Table 2.

All POx films were hydrophilic, their surface energy is in the range from 42.4 to 56.6 mJ m${}^{-2}$.

The thickness, hardness, and effective elastic modulus of POx films are given in Table 3.

The film thickness decreases with increasing substrate temperature at the deposition. This dependence is in agreement with the findings in previous experiments [22,23]. From the point of view of mechanical properties, the films showed polymer-like viscoelastic character. However, compared to common polymer materials (e.g., polycarbonate, polyethylene, polypropylene), they exhibited significantly higher hardness and effective elastic modulus (for example the hardness of polycarbonate is 0.18 GPa and its elastic modulus is around 3 GPa). The time dependent mechanical response of polymer materials may be characterized using the storage modulus $E^{\prime }$, loss modulus $E^{\prime \prime }$, and loss factor tanϕ. The storage modulus $E^{\prime }$ is the measure of the energy stored and recovered during the loading period, and the loss modulus $E^{\prime \prime }$ is the measure of the energy dissipated in the studied material during the loading period. The loss factor tanϕ is the measure of viscoelastic behavior of materials [27]. The studied POx films exhibited relatively low tanϕ values indicating a predominantly elastic behavior (low damping) of the film material, see Table 4.

**Figure 3:**
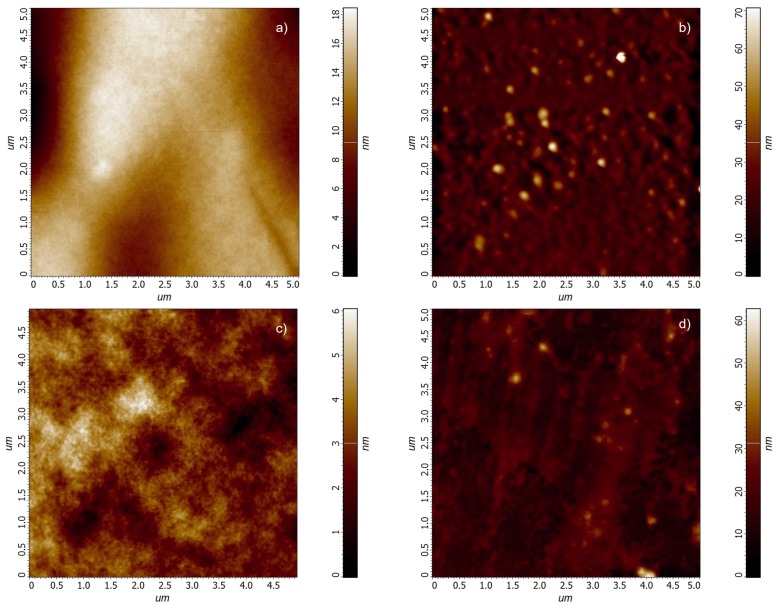
Atomic Force Microscope (AFM) images of POx films deposited at different substrate temperatures. (**a**) 60 ${}^{\circ }$C; (**b**) 90 ${}^{\circ }$C; (**c**) 120 ${}^{\circ }$C; (**d**) 150 ${}^{\circ }$C.

The AFM images of POx films are shown in Figure 3.

The film roughness values determined from AFM measurements are given in Table 5. Instead of the commonly used root mean square (RMS) roughness and average roughness values, the area peak-to-valley height $S_{t}$ and area peak density $Sd_{s}$ values [29] were added in order to illustrate the change of the film surface structure with the preparation temperature.

### 3.2. FTIR Analysis

**Figure 4:**
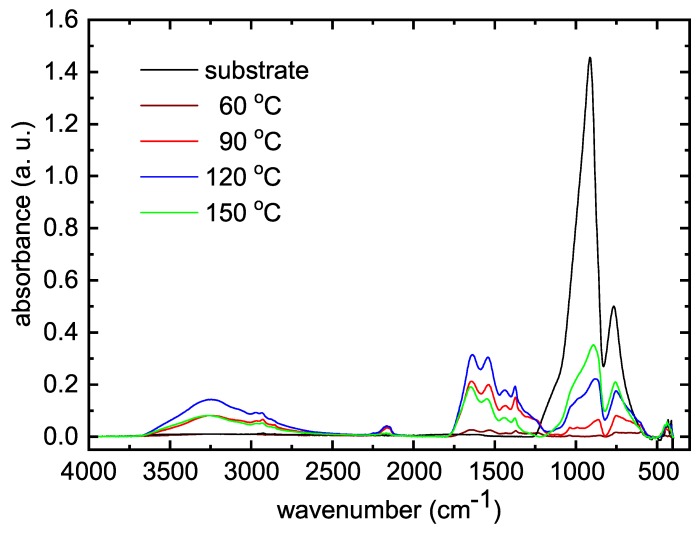
FTIR spectra of thin films deposited at different substrate temperatures.

The FTIR spectra of deposited POx thin films are shown in Figure 4.

Very low absorbance for the film deposited at substrate temperature of 60 ${}^{\circ }$C was caused by small film thickness. Broad absorption band in the range 3000–3600 cm${}^{-1}$ consists of several peaks belonging to OH, NH, and NH${}_{2}$ groups. The bands at 2950 cm${}^{-1}$, 1450 cm${}^{-1}$, and 1370 cm${}^{-1}$ are characteristic for vibrations of CH${}_{3}$ and CH${}_{2}$ groups. The band at 2170 cm${}^{-1}$ can be attributed to alkyne C≡C and/or isocyanate O=C=N and nitrile C≡N. Such chemical bonds are not present at traditional polymerization of oxazolines and they can be attributed to fragmentation and recombination of the oxazoline monomer during plasma polymerization. The band between 1790 cm${}^{-1}$ and 1590 cm${}^{-1}$ is characteristic for stretching vibration C=N bond constituting the oxazoline ring. Its presence in the IR spectrum indicates the presence of oxazoline rings in deposited films. The band around 1550 cm${}^{-1}$ belongs to N-H bonds. Finally, the bands below 1000 cm${}^{-1}$ belong to Si-O-Si or Si-O bonds from substrate glass.

### 3.3. Antibacterial Properties

**Figure 5:**
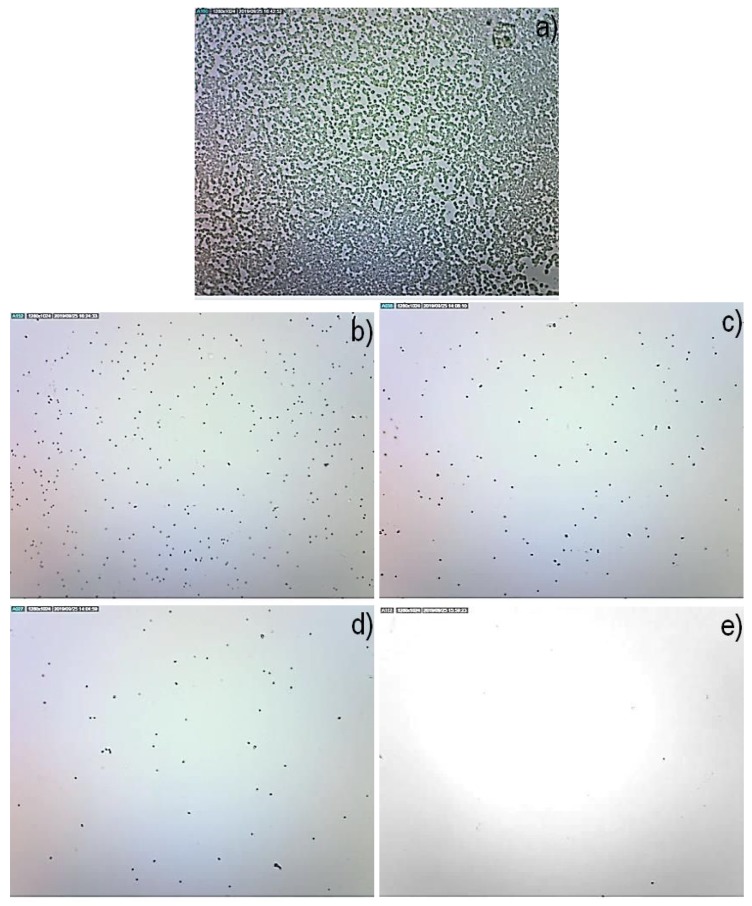
Images of films deposited at different substrate temperatures with bacteria *S. epidermidis*. (**a**) blank substrate; (**b**) 60 ${}^{\circ }$C; (**c**) 90 ${}^{\circ }$C; (**d**) 120 ${}^{\circ }$C; (**e**) 150 ${}^{\circ }$C.

Firstly, the antibacterial test was done using *S. epidermidis*, which was also used in previous studies [16,18]. Experiments were repeated twice using two identical series. It can be concluded that the results showed significant differences between the uncoated sample and samples with deposited films, see Figure 5. Bacteria on POx films were not able to form a biofilm. It can be observed in the microscopy image where only individual or small colonies are observed. In comparison, the bacteria growing on the untreated surface formed a biofilm. The possible explanations of antibiofouling properties can be found in a recent review [30].

**Figure 6:**
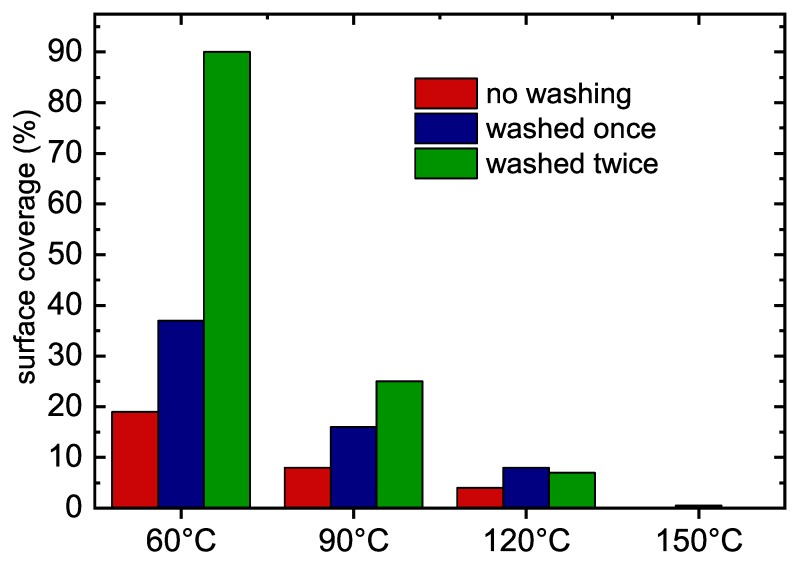
Bacteria *S. epidermidis* surface coverage area (percent) formed on POx films deposted at different substrate temperatures.

Before adding the micro-organism and incubation, some samples were washed once or twice with sterile Milli-Q water in order to test the stability of prepared POx films. The results are shown in Figure 6.

The films deposited at substrate temperatures of 60 ${}^{\circ }$C and 90 ${}^{\circ }$C are soluble in water and their antibiofouling properties decrease after their washing by water. On the other hand, the films deposited at substrate temperatures of 120 ${}^{\circ }$C and 150 ${}^{\circ }$C are not soluble in water and the washing does not decrease their antibiofouling properties significantly.

Secondly, the other antibacterial tests were done using *S. aureus* and *E. coli* according to the ISO 22196 procedure. Antibacterial activity against *S. aureus* and *E. coli* strains after 72 h of incubation time for the samples is listed in Table 6.

The reference substrate glass was open to both gram-positive and -negative bacterial contamination and did not perform any antibacterial effect, as expected. Nevertheless, counted viable gram-positive *S. aureus* level was found almost five times higher than the gram-negative *E. coli* strain. The oxazoline-based thin film deposited samples were highly active against both *S. aureus* and *E. coli* strains. The effect of the oxazoline-based thin film against *S. aureus* was slightly higher compared to *E. coli*, but the differences were negligible. Among them, as it can be seen in Table 6, the sample deposited at 150 ${}^{\circ }$C performed slightly lower antibacterial activity against both strains compared to other oxazoline-based thin film deposited samples, probably due to its higher bonding performance. However, it is also negligible, since the difference is extremely low. The antibacterial activity against *S. aureus* and *E. coli* depends on the cell-wall compositions of the bacterial strains and physicochemical characteristics with an efficiency and the releasing performance of the deposited oxazoline-based thin films. Therefore, the level of successfully deposited oxazoline-based thin films onto the glass substrate plays a critical role. Moreover, it strictly depends on the surface characteristics, such as wettability, roughness, charge density, and functionality.

### 3.4. Cytocompatibility Results

**Figure 7:**
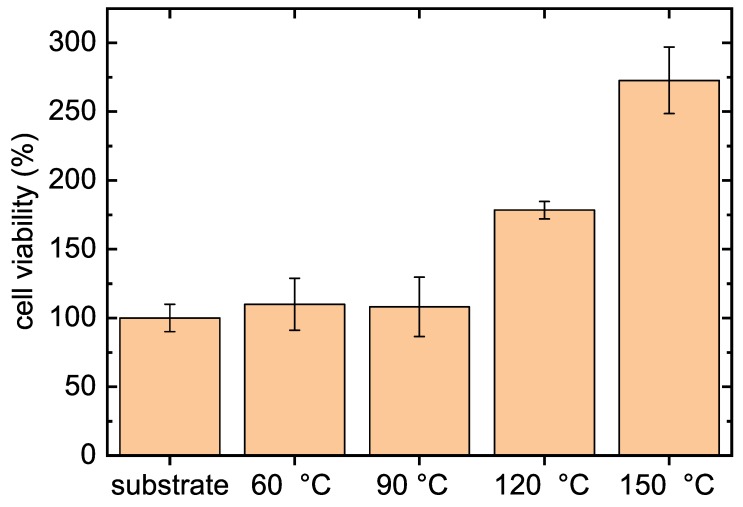
In vitro cytocompatibility results of tested POx films deposited at different substrate temperatures.

An in vitro cytocompatibility test was performed by mouse embryonic fibroblast cells (NIH/3T3) on the samples for 72 h and results are shown in Figure 7.

As can be seen from Figure 7, all types of modifications promote cell viability compared to blank glass substrate which was taken as reference. Films deposited at 60 ${}^{\circ }$C and 90 ${}^{\circ }$C exhibit almost identical cell viability as the reference. However, in the case of the film deposited at 120 ${}^{\circ }$C, the viability increased significantly when reaching the value of almost 180%. The highest fibroblasts cell viability was observed for the film deposited at 150 ${}^{\circ }$C when the value was more than 270% which signifies an excellent compatibility to the used cell substrate. The abovementioned results suggest that oxazoline-based thin films are affecting cell behavior, especially cell attachment. Moreover, with rising deposition temperature, the cell viability value is increasing.

## 4. Discussion

The oxazoline-based thin films were successfully deposited on glass substrates in atmospheric pressure dielectric barrier discharge. Nitrogen was used as the working gas for the discharge, 2-methyl-2-oxazoline vapors were admixed to the nitrogen flow and used as the monomer. This gas composition made it possible to obtain a homogeneous discharge, which led to the deposition of homogeneous thin films. To improve the film properties, it was necessary to increase the substrate temperature during the deposition. The deposited films were smooth and without any pinholes. The films deposited at substrate temperatures of 60 ${}^{\circ }$C and 90 ${}^{\circ }$C were soluble in water and they also did not exhibit increased cell viability compared to the blank glass substrate. It was found from FTIR spectra that some oxazoline rings are still present in the plasma polymer in contrast to classical oxazoline polymerization. The retention of the oxazoline ring is assumed to be highly beneficial for selected biomedical applications. On the other hand, some fragmentation and recombination of the oxazoline monomer was also observed in FTIR spectra. These findings are in agreement with the results of oxazoline plasma polymerization in low-pressure RF discharge [16]. However, higher N:C elemental ratio (0.93–0.81) was observed in films deposited in nitrogen APTD in this study, whereas lower N:C ratio (0.31–0.24) was observed at films deposited in RF discharge [16]. Nitrogen-rich surfaces are known for their excellent biocompatibility [31]. The best cell viability results were found at films deposited at substrate temperatures of 120 ${}^{\circ }$C and 150 ${}^{\circ }$C. Lower cell viability on films deposited at substrate temperatures of 60 ${}^{\circ }$C and 90 ${}^{\circ }$C can be explained by their dissolvability in aqueous solvents. All deposited films exhibited excellent antibacterial properties against all bacterial strains used for antibacterial tests. Deposited films could be used as coatings with antibacterial and antibiofouling properties for biomedical applications.

## Figures and Tables

**Table 1:** The elemental composition of films deposited at different substrate temperatures. The elemental composition is given in atomic %.

Element	60 ${}^{\circ }$C	90 ${}^{\circ }$C	120 ${}^{\circ }$C	150 ${}^{\circ }$C
C	43	43	47	48
N	40	41	42	39
O	17	16	11	13

**Table 2:** The contact angles for different liquids and surface free energy and its components of POx films deposited at different substrate temperatures.

Sample	Contact Angle (${}^{\circ }$)			Surface Free Energy (mJ/m${}^{2}$)		
	**CH** ${}_{2}$ **I** ${}_{2}$	**Glycerol**	**Water**	**Total**	**LW**	**AB**
substrate	59.8 ± 1.2	35.5 ± 2.0	33.4 ± 2.3	52.6 ± 1.0	28.7 ± 0.7	23.9 ± 2.0
60 ${}^{\circ }$C	40.5 ± 1.0	28.9 ± 0.8	10.0 ± 2.0	56.6 ± 0.7	39.4 ± 0.6	17.3 ± 1.2
90 ${}^{\circ }$C	40.8 ± 1.4	38.3 ± 0.5	16.1 ± 0.9	50.3 ± 0.9	39.2 ± 0.6	11.2 ± 1.4
120 ${}^{\circ }$C	60.8 ± 1.7	50.5 ± 1.8	40.0 ± 2.8	42.4 ± 1.9	28.1 ± 1.9	14.3 ± 2.8
150 ${}^{\circ }$C	60.6 ± 4.0	46.6 ± 1.7	21.9 ± 2.7	43.3 ± 1.8	27.8 ± 2.8	15.5 ± 4.0

**Table 3:** The film thickness, hardness, and effective elastic modulus of POx films deposited at different substrate temperatures.

Sample	Thickness (μm)	Hardness (GPa)	$E_{\mathrm{eff}}$ (GPa)
60 ${}^{\circ }$C	1.5 ± 0.2	0.70 ± 0.10	15 ± 1
90 ${}^{\circ }$C	1.7 ± 0.1	0.55 ± 0.05	11 ± 1
120 ${}^{\circ }$C	1.1 ± 0.1	0.55 ± 0.05	11 ± 1
150 ${}^{\circ }$C	0.6 ± 0.1	0.60 ± 0.05	15 ± 1

**Table 4:** The results of the nanodynamic indentation (nanoDMA) measurements: storage modulus $E^{\prime }$, loss modulus $E^{\prime \prime }$, and loss factor tanϕ of POx films deposited at different substrate temperatures.

Sample	$E^{\prime }$ (GPa)	$E^{\prime \prime }$ (GPa)	tanϕ
60 ${}^{\circ }$C	16 ± 2	0.50 ± 0.05	0.031 ± 0.005
90 ${}^{\circ }$C	11 ± 1	0.30 ± 0.04	0.027 ±0.006
120 ${}^{\circ }$C	12 ± 1	0.37 ± 0.05	0.031 ± 0.005
150 ${}^{\circ }$C	14 ± 1	0.48 ± 0.05	0.034 ± 0.005

**Table 5:** The film roughness of POx films deposited at different substrate temperatures. $S_{q}$—root mean square (RMS) roughness; $S_{a}$—average roughness; $S_{t}$—RMS roughness, area peak-to-valley height; $Sd_{s}$—area peak density.

Sample	$S_{q}$ (nm)	$S_{a}$ (nm)	$S_{t}$ (nm)	${\mathrm{Sd}}_{s}$ (μm${}^{-2})$
60 ${}^{\circ }$C	3.3	2.7	86.0	170
90 ${}^{\circ }$C	6.2	4.2	70.6	140
120 ${}^{\circ }$C	7.3	6.8	48.9	88
150 ${}^{\circ }$C	4.8	3.7	62.6	278

**Table 6:** Antibacterial activity results of studied POx films.

Sample	*S. aureus* (CFU/cm${}^{2}$)	*E. coli* (CFU/cm${}^{2}$)
substrate	$1.3\times 10^{6}$	$2.0\times 10^{5}$
60 ${}^{\circ }$C	<1	4.4
90 ${}^{\circ }$C	<1	1.1
120 ${}^{\circ }$C	<1	4.4
150 ${}^{\circ }$C	1.6	5.4
